# Supplementary material for: A Practical Application Primer on Cognitive Behavioral Therapy for Insomnia for Medical Residents
Source: MedEdPORTAL. 2019 Dec 13;15:10862. doi: 10.15766/mep_2374-8265.10862 (PMC7010204; doi:10.15766/mep_2374-8265.10862)
Supplement: Supplementary file 1 — A. CBT-I Presentation.ppt B. CBT-I Case Example.ppt C. CBT-I Reading List.doc D. Seminar Evaluation Form.docx [file mep-15-10862-s001.zip › _Educational_Summary_Report_10862.pdf]

MedEdPORTAL® The AAMC Journal of Teaching and Learning Resources

Original Publication Open Access

A Practical Application Primer on Cognitive Behavioral Therapy for Insomnia for Medical Residents

Yelena Chernyak, PhD*

*Corresponding author: ychernya@iupui.edu

Abstract

Introduction: Cognitive behavioral therapy for insomnia (CBT-I) is a well-established nonpharmacological intervention that is the gold standard treatment for insomnia. CBT-I has been utilized and empirically validated in many modalities, including group treatment, telemedicine, and primary care. Despite the wealth of evidence on its effectiveness, many medical providers, including those in primary care, where most insomnia complaints are raised, have limited exposure, knowledge, and resources to direct or implement this intervention. **Methods:** Medical educators from an academic medical center developed a module focused on teaching medical residents the techniques of CBT-I. The educational activity was an interactive 90-minute seminar that included a lecture followed by a case presentation illustrating the application of medical knowledge. A postseminar survey was used to evaluate the topic and content of the seminar. **Results:** In a survey of 32 primary care and psychiatry residents and sleep medicine fellows, 97% of respondents indicated that the topic of CBT-I should be included in the seminar series, and 84% indicated that the topic was of interest to them. Qualitative feedback underscored the relevance of this topic to trainees' clinical practice, as well as its underratedness. **Discussion:** The seminar on CBT-I was well received and viewed as a valuable tool in practicing medicine. The slides and vignettes provided enable replication of this workshop in other settings with medical learners who have a cursory knowledge of sleep medicine. The workshop is applicable to other health professionals, including medical students, nurses, social workers, and psychology trainees.

Keywords
Sleep, Insomnia, Sleep Initiation and Maintenance Disorders, Cognitive Behavioral Therapy, Evidence-Based Medicine/Knowledge Translation, Integrated Behavioral Health, Interprofessional Education, Lifestyle Medicine, Well-Being/Mental Health

Educational Objectives

By the end of this activity, learners will be able to:

1. Report increased awareness and knowledge of psychophysiological insomnia as a prevalent and treatable condition.
2. List the components of cognitive behavioral therapy for insomnia, including sleep hygiene, stimulus control, and sleep restriction.
3. Discuss clinical considerations of typical insomnia patients presenting to family medicine or psychiatry practice.

Citation:
Chernyak Y. A practical application primer on cognitive behavioral therapy for insomnia for medical residents. *MedEdPORTAL*. 2019;15:10862. https://doi.org/10.15766/mep_2374-8265.10862

Introduction

Insomnia is a very common problem, with an estimated prevalence of 10% in the general population,[1] increasing to 30% in primary care patients.[2] It is associated with poor quality of life and high health care costs.[3] Cognitive behavioral therapy (CBT) is a well-established form of psychotherapy that has been used to successfully treat many mental health conditions, including depression, anxiety, post-traumatic stress disorder, and others.[4] CBT has been tailored and applied to insomnia (CBT-I) in a treatment package that typically consists of stimulus control, sleep restriction, and cognitive therapy delivered individually or in groups over the course of six to 12 sessions. Randomized controlled trials have indicated that CBT-I is an effective treatment for insomnia, even when insomnia is chronic or comorbid with other medical or psychiatric conditions.[5-7] CBT-I is typically delivered by a trained psychologist, physician, or nurse, and has been shown to be effective in primary care settings using novel formats such as self-help, telehealth, and stepped care approaches, making it very versatile and accessible.[8-10]

Copyright © 2019 Chernyak. This is an open-access publication distributed under the terms of the Creative Commons Attribution license.

Traditional approaches to treating insomnia by physicians have been pharmacological interventions, shown to be comparable in the short term, but inferior in the long term, to CBT-I.[11,12] In 2016, the American College of Family Physicians issued a statement recommending that CBT-I be the first-line treatment for insomnia.[13] However, CBT-I remains underutilized, likely due to the lack of awareness and qualified treatment providers.[14] Medical residents would benefit from increased training in CBT-I to address the knowledge and skill gap in current de facto medical education. CBT-I needs to be a routine part of any physician's preparation to practice evidence-based medicine. A brief, implementable seminar on CBT-I that is appropriate for general medical trainees or practitioners would be an important addition to the medical education literature. This seminar should consist of a didactic component to fill the knowledge gap on insomnia and CBT-I, given the dearth of formal education on these topics in generalist settings. The didactic component should be followed by an interactive case presentation to illustrate application of knowledge with tools for assessment and treatment. Although there has been a self-learning module for medical students on insomnia published in *MedEdPORTAL*,[15] there is nothing currently available there on CBT-I.

Methods

This seminar was developed by an academic clinical health psychologist board certified in behavioral sleep medicine by the American Academy of Sleep Medicine. The purpose of this seminar was training advanced medical learners in CBT-I at the residency or fellowship level. Basic knowledge about insomnia or CBT generally was not a prerequisite to benefit from this workshop. This seminar was facilitated at multiple Indiana University School of Medicine residency didactic seminars, including those for the family medicine and psychiatry residencies. Workshop length was 90 minutes.

The seminar was delivered as part of a didactic seminar series in a large urban hospital. The audience primarily consisted of family medicine residents but also included psychiatry residents and sleep medicine fellows. A seminar accommodated six to 15 learners in any given session. Content was summarized in a PowerPoint presentation (Appendix A) that was projected on a large screen in a private meeting room. Copies of slides were provided to learners in advance. Learners were seated in a semicircle fashion to facilitate question-and-answer opportunities, as well as discussion.

A basic introduction to sleep psychophysiology based on Morin and Espie's *Insomnia: A Clinical Guide to Assessment and Treatment*[16] was presented. Components of CBT-I were based on Perlis, Jungquist, Smith, and Posner's *Cognitive Behavioral Treatment of Insomnia: A Session-by-Session Guide*.[17] Case examples from the presenter's clinical practice were weaved into the presentation five to 10 times throughout the presentation. Relevant cases were also elicited from the learners' active caseloads as illustrative examples. Three case vignettes that illustrated CBT-I treatment response (Appendix B) were reviewed at the conclusion of the presentation. Community referral resources and self-help resources for insomnia treatment were provided to the learners (Appendix C).

At the conclusion of the seminar, learners were asked to complete an anonymous survey on the content of the seminar (Appendix D). Surveys consisted of eight content areas evaluated on a 5-point Likert scale (1 = *strongly disagree*, 5 = *strongly agree*). Answers of strongly agree, agree, and neutral were considered favorable responses to the content areas evaluated. Respondents could also leave qualitative comments or suggestions as part of the survey. Trainees' participation in or responses to the survey were voluntary and did not affect evaluation of their performance by supervisors.

Results

Data were collected from 32 learners, 26 of whom were family medicine residents, two of whom were sleep medicine fellows, and four of whom were psychiatry residents at a large urban medical school. Approximately 60% of attendees ultimately completed surveys. At the conclusion of the seminar, all learners overwhelmingly felt that the educational objectives had been met. Nearly all learners (97%) felt that the topic of CBT-I should be included in the didactic series for their program, whereas the average rating of agreement with the statement "The topic should be included in the didactic series" was a 4.3 (based on a 5-point Likert scale). Most learners (84%) thought the presentation was interesting, with the average rating of agreement to a statement reflecting this being 4.1. Other positive responses to the seminar were reflected in consistently high ratings of agreement to the following statements: "The content was up to date" (4.3), "The presentation was organized" (4.3), "The A/V materials were well designed" (4.4), and "The speaker was easily understood" (4.2).

The average ratings of agreement with the statements "The presentation was appropriate for my level of expertise" and "The speaker allowed adequate time for questions" were 4.0 and 3.8, respectively, representing the two lowest-rated items of the survey (Table 1).

Copyright © 2019 Chernyak. This is an open-access publication distributed under the terms of the Creative Commons Attribution license.

Table 1. Cognitive Behavioral Therapy for Insomnia Didactic Series Quantitative Evaluation ($N = 32$)

	Strongly Disagree		Disagree		Neutral		Agree		Strongly Agree		
**Rating Items**	**No.**	**%**	**No.**	**%**	**No.**	**%**	**No.**	**%**	**No.**	**%**	**Avg.**
The topic should be included in the didactic series.	0	0.0	1	3.1	2	6.3	17	53.1	12	27.5	4.3
The content was up to date.	1	3.1	0	0.0	3	9.4	13	40.6	15	46.9	4.3
The presentation was interesting.	1	3.1	0	0.0	4	12.5	18	56.3	9	28.1	4.1
The presentation was appropriate for my level of expertise.	1	3.1	0	0.0	9	28.1	11	34.4	11	34.4	4.0
The presentation was organized.	1	3.1	0	0.0	2	6.3	16	50.0	13	40.6	4.3
The A/V materials were well designed.	1	3.1	0	0.0	0	0.0	16	50.0	15	46.9	4.4
The speaker was easily understood.	1	3.1	0	0.0	4	12.5	15	46.9	12	27.5	4.2
The speaker allowed adequate time for questions.	1	3.1	0	0.0	10	31.3	15	46.9	6	18.8	3.8

Qualitatively, learners provided written feedback on the survey. Sample comments included the following: “relevant to my practice; will improve patient care,” “great cases that were realistic and kept presentation interactive,” “interesting topic; very relevant and doctors are underprepared to deal with issue,” “great talk, underrated topic,” and “good topic; good info.” The author performed a thematic analysis of the qualitative comments from the workshops, which indicated that strengths of the seminar included the applied nature of the presentation, whereas recommendations for improvement focused on increased time to practice and role-play the strategies. Overall, these qualitative responses indicated that objectives to increase awareness, knowledge, and practice of CBT-I were met (Table 2).

Discussion

This seminar was designed to introduce CBT-I as the gold standard intervention for the treatment of psychophysiological insomnia to advanced medical learners. The seminar was well received and achieved its objectives, as demonstrated by overwhelmingly positive evaluations. Although designed primarily for resident physicians, the seminar can be utilized with a variety of health professionals, including medical students, nurses, and psychology trainees. Additionally, the seminar does not require prior knowledge of CBT, making it accessible to a wide audience. The fact that the lowest-ranked items on the survey referenced the appropriateness of the workshop for training level and adequate time for questions may indicate that further tailoring is required for various audiences.

An important factor for success was the interactive nature of the seminar, which included question-and-answer sessions and vignettes, engaging learners to think through typical cases, consider implementation in real-life practice, and receive real-time feedback. The greatest challenge of the workshop was managing the time dedicated to didactic overview compared to interactive discussion, which should not be allowed to overshadow delivery of critical didactic information.

Limitations of the seminar include that it was a single 90-minute session from which it is unknown whether learners retained new knowledge. CBT-I cannot be taught to proficiency in a single session. However, this primer is an excellent starting point for further education. Those learners who are interested could further their knowledge of CBT-I by reading published interventionist manuals, attending regional or national workshops, or selecting electives or fellowships to shadow practicing clinicians in the community. As CBT-I is a clinical intervention, hands-on practice with supervision is ultimately necessary to attain competency for direct care delivery.

Another limitation is the self-reported and time-limited nature of the feedback, which does not help determine what changes in future clinical practice actually ensued. In the future, it may be helpful to have a booster CBT-I practice session 1-3 months following the initial seminar. The objectives presented in this

Table 2. Thematic Analysis of Feedback

Element of Presentation	Theme of Feedback	Student Comments
Strengths	Applied nature	• “Great cases that were realistic and kept presentation interactive”
Topic selection	Relevant and underrated	• “Relevant to my practice; will improve patient care”<br>• “Interesting topic; very relevant and doctors are underprepared to deal with issue”<br>• “Great talk, underrated topic”
Improvement recommendations	Need to review more cases	• “More cases”<br>• “Increased time to practice”

Copyright © 2019 Chernyak. This is an open-access publication distributed under the terms of the Creative Commons Attribution license.

activity are not well aligned with the evaluation approach, which cannot precisely measure growth in knowledge or skills regarding CBT-I. The evaluation form that was used was designed to be simple and straightforward, and it prioritized minimizing respondent burden to increase completion rates. Because only about 60% of participants responded to the survey, there may have been a systematic difference between the perceptions of those participants who completed the surveys and those who did not. Moreover, despite the anonymous nature of the surveys, trainees may have been afraid to criticize those in a supervisory role.

In response to comments on the seminar, several adaptations have been made. The first is including a reading list of professional clinical resources for self-guided learning for those trainees who are more interested in adopting the skill set to deliver CBT-I themselves in their clinical practice. The second is increasing time for questions and in-session practice. The third effort, in progress, is outreach by the author to leadership in the medical school to expand training in this area across multiple professional levels.

With regard to dissemination, given the lack of board-certified behavioral sleep medicine practitioners or those with expertise in CBT-I specifically, finding facilitators to conduct similar seminars may be difficult. It may be necessary to seek expertise outside of typical academic medical centers or to invite speakers from outside geographical areas to deliver CBT-I seminars in areas without in-house expertise. As training in CBT-I becomes more commonplace in medical schools and residency programs, the hope is that this dearth of expertise will lessen, thereby benefiting the large number of patients who need this treatment. The implication of increasing medical education in CBT-I is significant. CBT-I is an underrated but highly effective and safe intervention at the forefront of evidence-based medicine. It is critical that medical providers are aware of this intervention for insomnia, a medical condition that effects more than one-third of individuals during their life span. Delivering this primer during medical education could help increase interest in this topic area and encourage further study of, dissemination of, and advocacy for this gold standard of care for insomnia, making CBT-I become routine. Appropriate referrals for CBT-I may help reduce the prescription of unnecessary, risky, and costly sleep aids that have not been shown to be highly effective or approved for long-term use. Although a subset of medical providers may be delivering CBT-I directly, for most medical providers, such as primary care physicians, sleep medicine specialists, pulmonologists, and psychiatrists, direct delivery of CBT-I can be conducted by specially trained colleagues such as psychologists or nurses. Therefore, trainings such as this primer can allow physicians to better identify psychophysiological insomnia, seek appropriate referrals, communicate with colleagues, monitor treatment progress, evaluate treatment response, and determine if next-line interventions for insomnia must be considered in the case of nonresponders.

Appendices

A. CBT-I Presentation.ppt

B. CBT-I Case Example.ppt

C. CBT-I Reading List.doc

D. Seminar Evaluation Form.docx

All appendices are peer reviewed as integral parts of the Original Publication.

Yelena Chernyak, PhD: Assistant Professor, Department of Psychiatry, Indiana University School of Medicine

Disclosures
None to report.

Funding/Support
None to report.

Ethical Approval
Reported as not applicable.

References

1. Ohayon MM. Epidemiology of insomnia: what we know and what we still need to learn. *Sleep Med Rev*. 2002;6(2):97-111. https://doi.org/10.1053/smrv.2002.0186
2. Hohagen F, Rink K, Käppler C, et al. Prevalence and treatment of insomnia in general practice: a longitudinal study. *Eur Arch Psychiatry Clin Neurosci*. 1993;242(6):329-336. https://doi.org/10.1007/BF02190245
3. Walsh JK. Clinical and socioeconomic correlates of insomnia. *J Clin Psychiatry*. 2004;65(suppl 8):13-19.
4. Butler AC, Chapman JE, Forman EM, Beck AT. The empirical status of cognitive-behavioral therapy: a review of meta-analyses. *Clin Psychol Rev*. 2006;26(1):17-31. https://doi.org/10.1016/j.cpr.2005.07.003
5. Standards of Practice Committee of the American Academy of Sleep Medicine. Practice parameters for the psychological and behavioral treatment of insomnia: an update. An American

Copyright © 2019 Chernyak. This is an open-access publication distributed under the terms of the Creative Commons Attribution license.

Academy of Sleep Medicine report. *Sleep*. 2006;29(11): 1415-1419. https://doi.org/10.1093/sleep/29.11.1415

6. Schutte-Rodin S, Broch L, Buysse D, Dorsey C, Sateia M. Clinical guideline for the evaluation and management of chronic insomnia in adults. *J Clin Sleep Med*. 2008;4(5):487-504.
7. Wu JQ, Appleman ER, Salazar RD, Ong JC. Cognitive behavioral therapy for insomnia comorbid with psychiatric and medical conditions: a meta-analysis. *JAMA Intern Med*. 2015;175(9): 1461-1472. https://doi.org/10.1001/jamainternmed.2015.3006
8. Ho FY-Y, Chung K-F, Yeung W-F, et al. Self-help cognitive-behavioral therapy for insomnia: a meta-analysis of randomized controlled trials. *Sleep Med Rev*. 2015;19:17-28. https://doi.org/10.1016/j.smrv.2014.06.010
9. Vincent N, Walsh K. Stepped care for insomnia: an evaluation of implementation in routine practice. *J Clin Sleep Med*. 2013;9(3):227-234. https://doi.org/10.5664/jcsm.2484
10. Edinger JD, Sampson WS. A primary care “friendly” cognitive behavioral insomnia therapy. *Sleep*. 2003;26(2):177-182. https://doi.org/10.1093/sleep/26.2.177
11. Morin CM, Colecchi C, Stone J, Sood R, Brink D. Behavioral and pharmacological therapies for late-life insomnia: a randomized controlled trial. *JAMA*. 1999;281(11):991-999. https://doi.org/10.1001/jama.281.11.991
12. Sivertsen B, Omvik S, Pallesen S, et al. Cognitive behavioral therapy vs zopiclone for treatment of chronic primary insomnia in older adults: a randomized controlled trial. *JAMA*. 2006;295(24): 2851-2858. https://doi.org/10.1001/jama.295.24.2851
13. Qaseem A, Kansagara D, Forciea MA, Cooke M, Denberg TD; for Clinical Guidelines Committee of the American College of Physicians. Management of chronic insomnia disorder in adults: a clinical practice guideline from the American College of Physicians. *Ann Intern Med*. 2016;165(2):125-133. https://doi.org/10.7326/M15-2175
14. Koffel E, Bramoweth AD, Ulmer CS. Increasing access to and utilization of cognitive behavioral therapy for insomnia (CBT-I): a narrative review. *J Gen Intern Med*. 2018;33(6):955-962. https://doi.org/10.1007/s11606-018-4390-1
15. Hawa R, Marcangelo M. Self-learning module (SLM): insomnia—an interactive learning module. *MedEdPORTAL*. 2014;10:9867. https://doi.org/10.15766/mep_2374-8265.9867
16. Morin CM, Espie CA. *Insomnia: A Clinical Guide to Assessment and Treatment*. New York, NY: Springer; 2004.
17. Perlis ML, Jungquist C, Smith MT, Posner D. *Cognitive Behavioral Treatment of Insomnia: A Session-by-Session Guide*. New York, NY: Springer; 2005.

Received: November 15, 2018
Accepted: August 4, 2019
Published: December 13, 2019

Copyright © 2019 Chernyak. This is an open-access publication distributed under the terms of the Creative Commons Attribution license.
