# Supplementary material for: A Practical Application Primer on Cognitive Behavioral Therapy for Insomnia for Medical Residents
Source: MedEdPORTAL. 2019 Dec 13;15:10862. doi: 10.15766/mep_2374-8265.10862 (PMC7010204; doi:10.15766/mep_2374-8265.10862)
Supplement: Supplementary file 1 — A. CBT-I Presentation.ppt B. CBT-I Case Example.ppt C. CBT-I Reading List.doc D. Seminar Evaluation Form.docx [file mep-15-10862-s001.zip › D. Seminar Evaluation Form.docx]

**Seminar Evaluation Form**

Title: _________________________________________ Presenter: __________________ Date:______

*Please circle your rating below for each item:*

|  | Strongly Disagree | Disagree | Neutral | Agree | Strongly Agree |
| --- | --- | --- | --- | --- | --- |
| 1. The topic should be included in the didactic series. | 1 | 2 | 3 | 4 | 5 |
| 1. The content was up to date | 1 | 2 | 3 | 4 | 5 |
| 1. The presentation was interesting. | 1 | 2 | 3 | 4 | 5 |
| 1. The presentation was appropriate for my level of expertise. | 1 | 2 | 3 | 4 | 5 |
| 1. The presentation was organized. | 1 | 2 | 3 | 4 | 5 |
| 1. The A/V materials were well designed. | 1 | 2 | 3 | 4 | 5 |
| 1. The speaker was easily understood. | 1 | 2 | 3 | 4 | 5 |
| 1. The speaker allowed adequate time for questions. | 1 | 2 | 3 | 4 | 5 |
| 1. The topic should be included in the didactic series. | 1 | 2 | 3 | 4 | 5 |

Comments:
