# Supplementary material for: A Practical Application Primer on Cognitive Behavioral Therapy for Insomnia for Medical Residents
Source: MedEdPORTAL. 2019 Dec 13;15:10862. doi: 10.15766/mep_2374-8265.10862 (PMC7010204; doi:10.15766/mep_2374-8265.10862)
Supplement: Supplementary file 1 — A. CBT-I Presentation.ppt B. CBT-I Case Example.ppt C. CBT-I Reading List.doc D. Seminar Evaluation Form.docx [file mep-15-10862-s001.zip › C. CBT-I Reading List.doc]

**COGNITIVE BEHAVIORAL THERAPY FOR INSOMNIA**

**READING RESOURCES**

| **Professional/Clinician Reading** | **Insomnia:  A Clinical Guide to Assessment and Treatment**  **Morin & Espie** |
| --- | --- |
|  | **Cognitive Behavioral Treatment of Insomnia: A Session-by-Session Guide**  **Jungquist, Posner, Perlis, & Smith** |
|  | Treating Sleep Disorders: Principles and Practice of Behavioral Sleep Medicine  Perlis & Lichstein |
|  | Behavioral Treatments for Sleep Disorders  Perlis, Aloia, Kuhn   |
| **Self-Help Reading** | **Overcoming Insomnia (there is a patient manual and therapist guide)**  **Treatment that Work Series** |
|  | **Cleveland Clinic Wellness Go to Sleep! Program**  [**http://www.clevelandclinicwellness.com/mind/BetterSleep/Pages/online-program-go-to-sleep.aspx**](http://www.clevelandclinicwellness.com/mind/BetterSleep/Pages/online-program-go-to-sleep.aspx) |
|  | **CBTI-Coach**  **App and Educational Module by VA & Stanford Collaboration** |
|  | **Say Goodnight to Insomnia** [**Gregg Jacobs**](http://www.amazon.com/exec/obidos/tg/detail/-/0805055487/ref=pd_bxgy_text_1//exec/obidos/search-handle-url/index=books&field-author=Jacobs%2C%252520Gregg/103-6813009-4331054) |

Web sites of interest: <http://www.sleepfoundation.org/publications.cfm>

Sleep hygiene principles: <http://medbroadcast.healthology.com/medbroadcast/16336.htm>
